# Changes in Nutrition-Intake Method and Oral Health through a Multidisciplinary Team Approach in Malnourished Older Patients Admitted to an Acute Care Hospital

**DOI:** 10.3390/ijerph19169784

**Published:** 2022-08-09

**Authors:** Hiroyuki Suzuki, Junichi Furuya, Kazuharu Nakagawa, Rena Hidaka, Ayako Nakane, Kanako Yoshimi, Yukue Shimizu, Keiko Saito, Yasuhiro Itsui, Haruka Tohara, Yuji Sato, Shunsuke Minakuchi

**Affiliations:** 1 Department of Gerodontology and Oral Rehabilitation, Graduate School of Medical and Dental Sciences, Tokyo Medical and Dental University (TMDU), 1-5-45 Yushima, Bunkyo-ku, Tokyo 113-8549, Japan; 2 Department of Geriatric Dentistry, School of Dentistry, Showa University, 2-1-1 Kitasenzoku, Ohta-ku, Tokyo 145-8515, Japan; 3 Department of Dysphagia Rehabilitation, Graduate School of Medical and Dental Sciences, Tokyo Medical and Dental University (TMDU), 1-5-45 Yushima, Bunkyo-ku, Tokyo 113-8549, Japan; 4 Department of Oral Health Sciences for Community Welfare, Graduate School of Medical and Dental Sciences, Tokyo Medical and Dental University (TMDU), 1-5-45 Yushima, Bunkyo-ku, Tokyo 113-8549, Japan; 5 Department of Nutrition Service, Tokyo Medical and Dental University Hospital, 1-5-45 Yushima, Bunkyo-ku, Tokyo 113-8549, Japan; 6 Medical Education Research and Development, Graduate School of Medical and Dental Sciences, Tokyo Medical and Dental University (TMDU), 1-5-45 Yushima, Bunkyo-ku, Tokyo 113-8519, Japan

**Keywords:** nutrition-intake method, oral health, swallowing function, nutrition support, multidisciplinary team approach

## Abstract

Malnourished older inpatients referred to a nutrition support team (NST) usually receive multidisciplinary oral health management during NST intervention. However, the effects of multidisciplinary oral health management on the nutrition-intake method and oral health in these patients remain unclear. This longitudinal study aimed to investigate the effects of NST-mediated multidisciplinary oral health management on the nutrition-intake methods, oral health, and the systemic and oral factors influencing the changes in the nutrition-intake method. A total of 117 inpatients (66 men, 51 women, mean age, 71.9 ± 12.5 years) who underwent NST-mediated multidisciplinary oral health management between April 2016 and July 2019 were enrolled. Demographic data and Functional Oral Intake Scale (FOIS), Dysphagia Severity Scale (DSS), and Oral Health Assessment Tool (OHAT) scores at the time of referral to the NST and completion of the NST intervention were evaluated. After multidisciplinary NST intervention, FOIS, DSS, and OHAT scores showed significant improvements (*p* < 0.001). Even after adjusting the results for systemic parameters, FOIS score improvement correlated positively with the length of NST intervention (*p* = 0.030) and DSS score improvement (*p* < 0.001) as well as OHAT score improvement (*p* = 0.047). NST interventions with multidisciplinary oral health management could improve the nutrition-intake method.

## 1. Introduction

Older adult inpatients are generally undernourished or at risk of malnutrition, and require adequate nutritional management to improve their general health status, prevent complications, and ensure treatment for all diseases [1]. Nutritional management by a multidisciplinary team of physicians, nurses, pharmacists, and dieticians is effective in improving patients’ quality of life (QOL) and reducing mortality, and this concept is widely practiced worldwide in the form of nutritional support teams (NSTs) [2,3,4]. Inpatients often receive parenteral or enteral nutrition based on factors such as the condition, accessibility, integrity, and function of their gastrointestinal tract and their anticipated length of therapy. Among the two methods of nutritional support, enteral nutrition has the added advantages of reduced incidence of nosocomial infections and shortened duration of hospitalization [5,6]. On the other hand, since oral ingestion is the most physiologically appropriate method of nutritional intake, the ultimate goal of an NST is the re-establishment of oral feeding to ensure the intake of the necessary nutrients. 

For the patients referred for NST intervention in an acute care hospital, the determination of oral intake capability is affected by their level of consciousness and swallowing function. Moreover, the determination of food form in patients who can feed orally gets affected by the swallowing function and the oral environment [7]. Additionally, in patients with dysphagia admitted to an acute care hospital, factors such as level of consciousness, activities of daily living (ADL), tongue and swallowing function, and molar occlusal support affect the method of nutritional intake [8]. Similarly, in patients admitted to chronic care hospitals, the nutrition-intake method is significantly correlated with the condition of the lips, tongue, gingiva/mucosa, saliva, and dentures, as in patients admitted to acute care hospitals [9]. Furthermore, the oral environment, particularly oral hygiene and prosthetic conditions, also has a significant effect on the appetite of older adult inpatients [10]. Therefore, maintenance of good oral health is essential for ensuring adequate nutritional support with oral intake in older inpatients.

However, several of these older patients, particularly those with diseases affecting their general health, impairments in consciousness, and poor oral management during hospitalization, show barriers to resuming oral intake due to poor oral hygiene, ill-fitting dentures, and reduced chewing and swallowing ability [11]. Inpatients in acute care hospitals referred to NSTs likewise show deteriorated oral environments, with tongue, saliva, and oral hygiene problems [12]. In such inpatients, multidisciplinary oral health management, including oral care by ward nurses, dysphagia rehabilitation by speech therapists, and specialized oral care and dental treatments by dental professionals, is effective [11]. However, the effects of multidisciplinary oral health management during the NST intervention period on the nutrition-intake method and oral health in malnourished older adult inpatients in acute care hospitals referred to NSTs remain largely unknown. 

Therefore, this longitudinal study assessed malnourished geriatric patients in an acute care hospital who underwent both multidisciplinary nutritional management and oral health management during the NST-intervention period. The changes in the nutrition-intake method, which was assessed using the Functional Oral Intake Scale (FOIS) [13], from start to completion of the NST intervention, were evaluated to assess the effects of NST with multidisciplinary oral health management on the nutrition-intake method. In addition, the changes in swallowing function and the oral environment, which were assessed using the Dysphagia Severity Scale (DSS) [14] and the Oral Health Assessment Tool (OHAT) [15], respectively, from start to completion of the NST intervention, were evaluated to investigate how oral health management affects swallowing function and the oral environment. Furthermore, this study analyzed the systemic and oral factors that affect the changes in the nutrition-intake method. 

## 2. Materials and Methods

### 2.1. Study Participants

This longitudinal study enrolled 348 inpatients (210 males and 138 females; mean age 66.9 ± 18.0 years) referred to the NST at Tokyo Medical and Dental University Hospital for nutritional management between April 2016 and July 2019 as potential participants. Patients who were less than 20 years old (*n* = 9), those who had not received nutritional management by NST (*n* = 84), and those who had not received multidisciplinary oral health management during the NST intervention (*n* = 138) were excluded from the study. Therefore, this study finally included 117 consecutive inpatients aged 20 years or older (66 men, 51 women; mean age, 71.9 ± 12.5 years) who underwent both multidisciplinary nutritional management and oral health management during the NST intervention period. The diet and nutrition-intake method of all the study participants were managed by the NST; the adjustments and monitoring were done in weekly multidisciplinary conferences. The NST team was composed of physicians, nurses, dentists, dental hygienists, dietitians, pharmacists, physical therapists, speech therapists, and other professionals. Oral health management for the participants was performed by dental professionals or ward nurses who received instructions from dental professionals. Oral health management in NST includes dental treatment such as caries treatment and denture treatment by dentists, professional oral care by dental hygienists, and oral care by ward nurses who receive oral health care instruction about the oral management techniques necessary for each patient’s condition from dental professionals. Participants with dysphagia underwent rehabilitation provided by speech therapists. Decisions to conclude the NST intervention were also made by multidisciplinary conferences based on the assessment of improvement in nutritional status. The use of anonymized clinical data was explained in a document posted on the Tokyo Medical and Dental University Hospital website to ensure that patients could choose to decline participation by the opt-out method. This study was approved by the Dental Research Ethics Committee of Tokyo Medical and Dental University (approval no. D2016-077). 

### 2.2. Study Parameters 

This study evaluated the nutrition-intake method, swallowing ability, and oral environment at the time of referral and completion of the NST intervention. These assessments were made by two dentists who underwent sufficient calibration to ensure matching assessment criteria before starting the study. The nutrition-intake method was evaluated using the Functional Oral Intake Scale (FOIS), which categorizes nutrition intake from level 1 (“nothing by mouth”) to 7 (“total oral diet with no restrictions”) [13]. Swallowing function was assessed by the Dysphagia Severity Scale (DSS), in which the severity of dysphagia is rated from level 1 (“saliva aspiration”) to 7 (“within normal limits”) [14]. The oral environment was appraised using the Oral Health Assessment Tool (OHAT), in which the lip, tongue, gingiva/mucosa, saliva, remaining teeth, dentures, oral hygiene, and dental pain are rated from 0 to 2 [15]. The total score ranges between 0 and 16 points, and higher scores indicate a worse oral environment. 

### 2.3. Other Variables

Demographic data of the study participants, i.e., age, sex, duration of hospitalization (days), duration of NST intervention, BMI, general condition, level of consciousness, level of independence, and type of professional who performed the oral health management for the patient, were extracted from the medical records and NST conference data. For the evaluation of the patient’s general condition, their primary disease and comorbidities were scored using the Charlson Comorbidity Index (CCI) [16]. The level of consciousness was assessed using the Japan Coma Scale (JCS), where a score of 0 indicated alertness, and other states were categorized as “aroused (I)”, “arousable with some stimulation (II)”, and “unarousable by any stimulation (III) [17]”. Level of independence was evaluated by the Performance Status (PS), with scores ranging from 0 (able to perform all activities of daily living without problems or restrictions) to 4 (unable to move at all or perform any activities of daily living and spending the entire day in bed or in a chair) [18]. 

### 2.4. Statistical Analyses

A clinical statistical analysis of the participants’ basic demographics was performed. Furthermore, the Wilcoxon signed-rank test was used to compare participants’ nutrition-intake methods, swallowing ability, and oral environments at the time of NST referral and at the completion of the NST interventions. To further evaluate the factors affecting the changes in the nutrition-intake method mediated by the multidisciplinary oral health management in NST, a multiple-regression analysis was performed with changes to the FOIS score after NST intervention (FOIS at the completion of NST intervention—FOIS at referral to the NST) as the objective variable, and age, sex (male = 0, female = 1), length of NST intervention (days), BMI, CCI, JCS score, PS score, change in DSS score at the completion of the NST intervention (DSS score at the completion of NST intervention—DSS score at referral for NST intervention), change in OHAT score at the end of the NST intervention (total OHAT score at the completion of NST intervention—total OHAT score at referral for NST intervention), and type of professional performing oral health management (0 = oral management by ward nurse, 1 = oral management by dental professional) as the explanatory variables. Higher values of changes in FOIS and DSS scores and lower values of changes in OHAT scores indicated better improvement. SPSS Version 28.0 (IBM Japan, Tokyo, Japan) was used for all statistical analyses, and *p* < 0.05 was defined as statistically significant. 

## 3. Results

### 3.1. Participant Characteristics 

The basic demographic data of the study participants are shown in Table 1. The age distribution of the study participants was as follows: 28 participants were aged 64 years or younger (23.9%), 33 were between 65–74 years (28.2%), and 56 were 75 years and older (47.9%), indicating a high percentage of older inpatients. The durations of hospitalization and NST intervention varied widely. The mean durations of hospitalization and NST intervention were 76.2 ± 64.0 and 33.1 ± 35.3 days, respectively. Participants in this study were patients with a wide range of diseases, including stroke, heart disease, diabetes, kidney disease, and malignant tumors, who were hospitalized for treatment and were referred to the NST for nutritional management. Many participants had a relatively good level of consciousness but showed restricted activities of daily living, and the majority spent 50% or more of their days in bed or in a chair. Altogether, 53.8% of the participants underwent oral health management by nurses who were instructed on oral management by dental professionals. 

### 3.2. Changes in the Nutrition-Intake Method, Swallowing Ability, and Oral Environment Associated with Multidisciplinary Oral Health Management

The results of the nutrition-intake method (FOIS), swallowing function (DSS), and oral environment (OHAT) at the time of referral and completion of the NST intervention are shown in Table 2. On assessment of the nutrition-intake method, the mean FOIS score improved significantly from 2.6 ± 2.1 to 3.4 ± 2.5 after NST intervention (*p* < 0.001). At the time of referral to the NST, 55.6% of the participants were incapable of oral intake, whereas only 29.1% of participants were able to have sufficient nutrition by oral intake alone. However, at the completion of the intervention, the percentage of participants incapable of oral intake decreased to 41.9% and that of patients able to have sufficient nutrition by oral intake alone increased to 45.2%. 

With regard to swallowing ability, the mean DSS score improved significantly from 3.3 ± 1.9 to 3.7 ± 2.0 after the NST intervention (*p* < 0.001). Moreover, 68.3% of the patients showed aspiration at the time of referral to the NST, which decreased to 60.7% at the time of completion of the intervention. Likewise, the mean total OHAT score, an indicator of the oral environment, improved significantly from 6.1 ± 3.0 at the start of the NST intervention to 3.6 ± 2.7 at the completion of the intervention by implementation of multidisciplinary oral health management (*p* < 0.001). The subscale items, with the exception of the remaining teeth, also improved significantly. 

### 3.3. Factors Affecting the Changes in Nutrition-Intake Method Elicited by Multidisciplinary Oral Health Management 

The results of the multiple-regression analysis, with changes in FOIS scores during the NST intervention as the objective variable, are shown in Table 3. Even after adjusting for age, sex, nutritional status, general conditions, and type of professional who performed the oral health management, the changes in the FOIS score mediated by the multidisciplinary oral health management of the NST correlated positively and significantly with the length of the NST intervention (*p* = 0.030) and DSS score changes (*p* < 0.001), and negatively and significantly with OHAT score changes (*p* = 0.047). 

## 4. Discussion

This study revealed that NST-mediated multidisciplinary oral health management comprising of oral care, dental treatment, and dysphagia rehabilitation might improve the nutrition-intake method and potentially improve swallowing ability and the oral environment. Furthermore, the improvements in the nutrition-intake method with multidisciplinary oral health management were associated with the length of NST intervention, degree of improvement in swallowing ability, and the degree of improvement in the oral environment, independently of age, sex, nutritional state, systemic disease, level of consciousness, ADL, and the type of professional providing the oral health management. The clinical importance of this study is that it suggests that multidisciplinary oral health management by NST might contribute to improved nutritional management (e.g., by re-establishing oral intake) in acute care hospital patients whose oral conditions are deteriorating [12]. Furthermore, expert interventions by a dentist and adequate oral health management guidance to nurses and other professionals are important aspects of a multidisciplinary NST [12]. 

Malnourishment status and the associated risks increase relatively early in inpatients at acute care hospitals [19]. Therefore, nutritional management should be implemented as early as possible for the treatment of the primary disease as well as for preventing complications. Parenteral nutrition or tube feeding, which ensures nutritional intake, is often selected to meet this goal in acute care hospital inpatients. Nevertheless, the method chosen by an appropriate multidisciplinary assessment of the patient’s general oral conditions and functions may differ from the originally chosen nutrition-intake method [7,8]. These findings highlight the importance of selecting a nutrition-intake method tailored to the patient’s abilities, after thorough assessment by a multidisciplinary team, in order to avoid unnecessary restrictions to oral intake and, thereby, improve and maintain inpatient QOL. One of the most important roles of multidisciplinary NST is to promote a nutrition-intake method adapted to the patient’s abilities after obtaining an accurate assessment of the patient’s condition, including that of their oral environment. This study showed a significant improvement in the nutrition-intake method after the NST intervention, which was due to the selection of an appropriate nutrition-intake method proposed to the attending physician by the NST after thorough patient assessment. 

Dysphagia and poor oral health, often observed in older inpatients, are associated with low QOL, refusal of oral intake, malnutrition, and mortality [7,20,21,22,23]. However, countermeasures by a multidisciplinary team may be effective [11]. For example, the intervention by speech therapists in the examination of swallowing dynamics in patients with dysphagia and swallowing rehabilitation, and of the physician, dentist, and dietician in providing nutritional support, such as proposing a texture-modified diet, can improve dysphagia [24,25]. While oral health management by dental professionals can improve the oral environment, oral health interventions implemented by ward nurses instructed by dental professionals can also improve the oral environment, particularly the oral hygiene, of inpatients [26]. 

The median DSS of the participants in this study was 3, and approximately 70% of the study population reported aspiration. The study population showed a higher rate of dysphagia than that in a previously reported study involving acute care hospital inpatients [27]. Likewise, the patients had a poor oral condition, with a median total OHAT score of 6, which was poorer than previously reported scores in inpatients [11,12]. However, multidisciplinary oral health management improved the DSS, and the total OHAT scores significantly reduced to 4 and 3, respectively, although aspiration persisted in approximately 60% of the participants. The results of the previous studies and the present study show that swallowing function and oral environment of inpatients can be improved by NST. Moreover, these studies also emphasize the importance of the active implementation of an NST. 

In this study, changes to the nutrition-intake method were associated with the duration of the NST intervention, changes to swallowing function, and changes to the oral environment, independent of age, sex, nutritional status, systemic disease, level of consciousness, ADL, and the type of professional that performed the oral health management interventions. This suggests that improvements in swallowing function and the oral environment were associated with improvements in the nutrition-intake methods. The frequency of implementation of nutritional management affected the nutritional status and the patient’s oral intake ability [25]. The association between improvements in the nutrition-intake method and duration of NST intervention was attributed to the fact that longer durations of NST interventions would increase opportunities for evaluating and re-evaluating the patient’s general condition and oral environment and reconsidering the appropriate nutrition-intake method. Yoshimi et al. reported that dental interventions, such as prosthetic treatment; tooth extraction; and specialized oral care, by dental professionals in patients with aspiration pneumonia during the hospital stay, to improve the oral environment and function, increased the likelihood of re-establishing oral intake after discharge [28]. Aoyagi et al. reported that in patients with stroke, a one-point improvement in DSS scores and a one-point improvement in the total OHAT score were associated with seven- and 1.2-fold improvements in the nutrition-intake method, respectively [29]. Therefore, improvements in swallowing function as well as the oral environment in inpatients could facilitate the selection of less-restrictive nutrition-intake methods. Our study also seemed to mirror their outcomes. 

This study had several limitations. First, it only evaluated parameters of the oral environment but did not evaluate oral function parameters such as tongue mobility. Oral function is known to reduce with age, and tongue and lip mobility are especially associated with the nutrition-intake method [21,30]. Additionally, evaluation of oral function would have allowed the identification of factors that affect NST-related changes in nutrition-intake method and in conceiving methods of interventions aimed to effectively improve oral functions in patients referred for NST interventions. Therefore, the oral function of inpatients should be investigated further in future studies. Second, the lack of QOL assessment of the participants was also one of the limitations of this study. Although recent studies have reported that nutritional management by a multidisciplinary team may improve the QOL of patients, it is still unclear how changes in nutrition-intake method and changes in the oral environment are related to these improvements in quality of life. If QOL assessments had been conducted, it may have been possible to determine the impact of the changes in the oral environment on QOL through multidisciplinary nutritional management with oral health management. Third, this study included only patients who received both multidisciplinary nutritional management and oral management during the NST intervention period. If patients who did not receive oral management, instead only receiving nutritional management during the NST intervention period, were followed up as a control group, it is highly possible that the effect of oral management could have been clarified, which is also a limitation. Finally, this was a single-center, longitudinal study; since the frequency and methods of the interventions provided by the NST intervention period were not controlled, it is not possible to accurately define the NST multidisciplinary oral health management methods based on the results of this study. However, the general conditions and oral environments of the inpatients referred to the NST were diverse, making it unrealistic to assume that a one-size-fits-all oral health management method would work for all patients. In clinical practice, dental professionals, nurses, and other multidisciplinary staff should work together to create tailored oral health management methods for individual patients. The importance and efficacy of such tailored oral health management methods, as shown by our data, demonstrate the clinical significance of this study. 

## 5. Conclusions

This study revealed that NST interventions with multidisciplinary oral health management might improve the nutrition-intake method, and that these improvements were associated with the duration of the NST intervention and the degree of improvement in swallowing and the oral environment, independent of age, sex, nutritional status, general condition, consciousness level, ADL, and the type of professional providing the oral health management. Therefore, the findings suggested that the oral care, dental treatments, and dysphagia rehabilitation performed through multidisciplinary cooperation by the NST are important for improving swallowing ability and the oral environment. 

## Figures and Tables

**Table 1:** Basic demographic characteristics of the study participants.

		Mean ± SD	Median	*n*	%
Age		71.9 ± 12.5	74	117	100.0
Sex	Male			66	56.4
	Female			51	43.6
Height (cm)		158.5 ± 9.3	158.7	117	100.0
Weight (kg)		50.6 ± 13.6	48.3	117	100.0
BMI (kg/m^2^)		20.0 ± 4.5	19.6	117	100.0
Length of hospitalization (days)		76.2 ± 64.0	51	117	100.0
Length of NST intervention (days)		33.1 ± 35.3	22	117	100.0
CCI		2.8 ± 2.5	2	117	100.0
Level of consciousness (JCS score)	0			33	28.2
	I			67	57.3
	II			10	8.5
	III			7	6.0
Independence (PS)	0			2	1.7
	1			9	7.7
	2			20	17.1
	3			37	31.6
	4			49	41.9
Type of professional performing the oral management	Ward nurses			63	53.8
	Dental professionals			54	46.2

Abbreviations: SD, standard deviation; BMI, body mass index; NST, nutritional support team; CCI, Charlson Comorbidity Index; JCS, Japan Coma Scale; PS, performance status.

**Table 2:** Changes in the nutrition-intake method, swallowing ability, and oral environment elicited by NST intervention including multidisciplinary oral health management.

		At Referral to NST				At Completion of NST				
		Mean ± SD	Median	*n*	%	Mean ± SD	Median	*n*	%	*p*-Value
FOIS score		2.6 ± 2.1	1	117		3.4 ± 2.5	2	117		<0.001 *
	1			65	55.6			49	41.9	
	2			11	9.4			10	8.5	
	3			7	6.0			5	4.3	
	4			5	4.3			4	3.4	
	5			7	6.0			11	9.4	
	6			15	12.8			19	16.2	
	7			7	6.0			19	16.2	
DSS score		3.3 ± 1.9	3	117		3.7 ± 2.0	4	117		<0.001 *
	1			28	23.9			20	17.1	
	2			24	20.5			26	22.2	
	3			13	11.1			7	6.0	
	4			15	12.8			18	15.4	
	5			17	14.5			17	14.5	
	6			17	14.5			17	14.5	
	7			3	2.6			12	10.3	
OHAT score		6.1 ± 3.0	6	117	100	3.6 ± 2.7	3	117	100	<0.001 *
	Lip	0.6 ± 0.7	1			0.3 ± 0.5	0			<0.001 *
	Tongue	0.9 ± 0.7	1			0.6 ± 0.5	1			<0.001 *
	Gingiva/mucosa	0.6 ± 0.7	0			0.4 ± 0.6	0			<0.001 *
	Saliva	1.1 ± 0.7	1			0.6 ± 0.6	1			<0.001 *
	Remaining teeth	0.5 ± 0.8	0			0.5 ± 0.8	0			0.317
	Dentures	0.9 ± 1.0	0			0.7 ± 1.0	0			0.002 *
	Oral hygiene	1.0 ± 0.8	1			0.4 ± 0.6	0			<0.001 *
	Dental pain	0.4 ± 0.7	0			0.2 ± 0.5	0			<0.001 *

* *p* < 0.05 at the time of referral to NST vs. at the time of completion of NST; Wilcoxon signed-rank test. Abbreviations: NST, nutritional support team; DSS, Dysphagia Severity Scale; FOIS, Functional Oral Intake Scale; SD, standard deviation; OHAT, Oral Health Assessment Tool.

**Table 3:** Factors affecting changes in FOIS scores after the NST intervention including multidisciplinary oral health management.

Independent Variables	B	SE	95% Confidence Interval of Estimate	β	*p* Value	Variance Inflation Factor
Age	−0.003	0.012	−0.027 to 0.022	−0.018	0.822	1.093
Sex	0.361	0.312	−0.257 to 0.978	0.091	0.250	1.123
Duration of NST intervention	0.009	0.004	0.001 to 0.018	0.170	0.030 *	1.074
BMI	0.003	0.034	−0.064 to 0.069	0.006	0.940	1.088
CCI	−0.064	0.061	−0.185 to 0.057	−0.082	0.299	1.108
JCS	−0.322	0.219	−0.756 to 0.112	−0.128	0.144	1.353
PS	0.015	0.170	−0.322 to 0.353	0.008	0.928	1.432
Change in DSS score after intervention	0.944	0.144	0.658 to 1.230	0.537	<0.001 *	1.213
Change in OHAT score after intervention	−0.153	0.076	−0.304 to −0.002	−0.166	0.047 *	1.227
Type of professional performing oral management	−0.380	0.321	−1.016 to 0.256	−0.097	0.239	1.202

Abbreviations: FOIS, Functional Oral Intake Scale; NST, nutritional support team; BMI, body mass index; CCI, Charlson Comorbidity Index; JCS, Japan Coma Scale; PS, performance status; DSS, Dysphagia Severity Scale; OHAT, Oral Health Assessment Tool. Multiple R = 0.642; R^2^ = 0.412; *p* < 0.001. B, partial regression coefficient; SE, standard error; β, standardized partial regression coefficient; change in FOIS score after intervention: FOIS at the completion of NST intervention—FOIS at referral to the NST, higher values of the change in FOIS scores indicated better improvement; sex: Male = 0, Female = 1; change in DSS score after intervention: DSS score at the completion of NST intervention—DSS score at referral for NST intervention, higher values of the change in DSS scores indicated better improvement; change in OHAT score after intervention: total OHAT score at the completion of NST intervention—total OHAT score at referral for NST intervention, lower values of the change in OHAT scores indicated better improvement; type of professional performing oral management: ward nurses = 0, dental professionals = 1. All other independent variables were used as continuous variables. *: *p* < 0.05, multiple-regression analysis.

## Data Availability

Not applicable.
